# Medical malpractice claims in laparoscopic gynecologic surgery: a Dutch overview of 20 years

**DOI:** 10.1007/s00464-017-5624-8

**Published:** 2017-06-20

**Authors:** Evelien M. Sandberg, Esmée M. Bordewijk, Désirée Klemann, Sara R. C. Driessen, Andries R. H. Twijnstra, Frank Willem Jansen

**Affiliations:** 1 Department of Gyaecology, Section Minimally Invasive Surgery, Leiden University Medical Centre, PO Box 9600, 2300 RC Leiden, The Netherlands; 2 Department of Gynecology, Academic Medical Centre, Amsterdam, The Netherlands; 3 Department of Gynecology, Maastricht University Medical Centre, Maastricht, The Netherlands; 4 Department Bio Mechanical Engineering, Delft University of Technology, Delft, The Netherlands

**Keywords:** Medical claims, Laparoscopic gynecologic surgery, Delayed diagnosis, Laparoscopic entry-related injuries, Bowel and ureter complications

## Abstract

**Background:**

The success of newly introduced surgical techniques is generally primarily assessed by surgical outcome measures. However, data on medical liability should concomitantly be used to evaluate provided care as they give a unique insight into substandard care from patient’s point of view. The aim of this study was to analyze the number and type of medical claims after laparoscopic gynecologic procedures since the introduction of advanced laparoscopy two decades ago. Secondly, our objective was to identify trends and/or risk factors associated with these claims.

**Methods:**

To identify the claims, we searched the databases of the two largest medical liability mutual insurance companies in The Netherlands (MediRisk and Centramed), covering together 96% of the Dutch hospitals. All claims related to laparoscopic gynecologic surgery and filed between 1993 and 2015 were included.

**Results:**

A total of 133 claims met our inclusion criteria, of which 54 were accepted claims (41%) and 79 rejected (59%). The number of claims remained relatively constant over time. The majority of claims were filed for visceral and/or vascular injuries (82%), specifically to the bowel (40%) and ureters (20%). More than one-third of the injuries were entry related (38%) and 77% of the claims were filed after non-advanced procedures. A delay in diagnosing injuries was the primary reason for financial compensation (33%). The median sum paid to patients was €12,000 (500–848,689). In 90 claims, an attorney was defending the patient (83% for the accepted claims; 57% for the rejected claims).

**Conclusion:**

The number of claims remained relatively constant during the study period. Most claims were provoked by bowel and ureter injuries. Delay in recognizing injuries was the most encountered reason for granting financial compensation. Entering the abdominal cavity during laparoscopy continues to be a potential dangerous step. As a result, gynecologists are recommended to thoroughly counsel patients undergoing any laparoscopic procedure, even regarding the risk of entry-related injuries.

**Electronic supplementary material:**

The online version of this article (doi:10.1007/s00464-017-5624-8) contains supplementary material, which is available to authorized users.

Safely introducing new technologies in the surgical field is challenging, particularly for highly advanced procedures. In contrast to the introduction of new drugs, (surgical) techniques and devices may not be introduced prior to extensive evaluation and their true impact can often only be appreciated over time [1–3]. Consequently, recent reports and studies from different medical fields have recommended systematic evaluations of efficacy and safety of every newly introduced (surgical) technique or instrument [1–3]. The success of new technology is generally primarily assessed by clinical outcome measures. In an era where Value-Based Health Care is being broadly implemented, other source of information should also be concomitantly used to evaluate provided care. One interesting and complementary source of information is data on medical liability [4]. Even though litigation climate varies among countries and not every claim is the consequence of an adverse event, these data provide a unique insight into incidents judged by patients as substandard care [5].

Over the past two decades, laparoscopic surgery has been rapidly implemented in many countries [6]. Although the minimally invasive technique is still advancing, its introduction has definitely changed our daily surgical practice. Minimally invasive surgery has even been described as the most important revolution in surgical technique since the early 1900s [7]. In the field of gynecology, advanced laparoscopic surgery has been widely introduced two decades ago. Understanding the reasons for filing claims, especially in new (surgical) fields, should be part of the evaluation process to improve care. As a result, we aimed in this present study to analyze the medical liability claims of laparoscopic gynecologic procedures in The Netherlands since the broad introduction of (advanced) laparoscopy two decades ago. Secondly, our objective was to identify trends and/or risk factors associated with these claims.

## Materials and methods

### Selection criteria

To identify the medical claims of laparoscopic gynecologic surgery, we searched the databases of the two largest medical liability mutual insurance companies in The Netherlands (MediRisk and Centramed). The search terms used were ‘gynecology’ and ‘laparoscopy’ and all claims concerning laparoscopic gynecologic surgery were included up to 1st of January 2016. Claims were available from 1993 for MediRisk and 1995 for Centramed, the founding years of the companies. The study was exempted from Institutional Review Board approval.

MediRisk and Centramed currently cover together 87 of the 91 Dutch hospitals (95.6%). The insured hospitals are teaching and non-teaching hospitals and Centramed specifically insures six of the eight Dutch academic hospitals.

To evaluate the impact of laparoscopic gynecological surgery, we exclusively included claims related to injuries and/or technological failures. We excluded claims regarding unwanted pregnancies after failed laparoscopic sterilization and claims concerning intra-uterine procedures (e.g., hysteroscopy and intra-uterine device placement).

Both claims of accepted and rejected cases were included. An accepted case signifies that the medical insurance company recognizes that the given care was suboptimal and that the adverse event could have been avoided. These patients are being financially compensated for the caused damage. A rejected case means that, although an adverse event may have occurred, no medical malpractice was observed. As such, no pay-outs were granted for those cases. Also, both open and closed claims were included in the present study. The ‘open claims’ were only included if the verdict on liability was available when chart review was performed (October 2016).

### Data extraction

The medical and legal charts of all selected claims were reviewed at the insurance company offices. The following data were extracted: (1) description of the incident including the moment the incidence was discovered, (2) legal information (liability, the presence of an attorney, time frame, costs, and pay-outs), (3) patient characteristics [age and BMI (kg/m^2^) at initial procedure, previous surgeries, health care-related job, type of hospital (teaching, non-teaching)], and (4) surgical procedures (classified according to the European Society for Gynecological Endoscopy (ESGE) [8] and complications. Complications were defined following the internationally recognized classification of the Dutch Society of Obstetrics and Gynecology (NVOG) [9]. Each complication was further subcategorized into four categories: (A) temporary disability, no re-operation required; (B) disability resolved after re-operation; (C) permanent disability; and (D) death. Detailed information on the ESGE classification for laparoscopic procedures and the NVOG classification for complications is available in the Supporting Information (Table S1 and Table S2).

### Statistics

Data were analyzed using SPSS version 23 for Windows. Collected data were summarized and outliers were reviewed. Continuous data were presented as median with minimum and maximum and categorical data as frequency and percentages.

## Results

### Claim selection

Over the study period, 328 claims were identified (Supporting Information, Figure S1). A total of 146 claims (44.5%) did not meet our inclusion criteria and were excluded. In addition, 49 claims (15%) were not available as their files had been destroyed or could not be found in the archives anymore (29 for MediRisk and 20 for Centramed). A total of 133 claims were eventually included in our study (119 from MediRisk and 14 from Centramed).

Of these 133 claims, 79 were rejected by the medical insurance company (59.3%) and 54 were accepted (40.6%), of which 20 with an amicable settlement. A total of sixteen claims were still open at the time of our study but as their verdicts were known, they were included in the analysis. These claims had not been closed yet, as for the rejected claims (*n* = 11) an appeal had been made and for the accepted claims (*n* = 5) the amount of pay-outs was still being negotiated.

### Patient and surgical characteristics

Table 1 depicts the baseline characteristics of the women filing a claim and their indication for surgery. Twenty-one of the women filing a claim (21.6%) were working themselves in the medical sector. During the study period, 63 of the 87 hospitals (72.4%) had at least one claim and the number of claims per hospital varied, with a maximum of six claims. Slightly more claims were filed by patients treated in teaching hospitals compared to the non-teaching hospitals (55.8 vs. 44.2%).

**Table 1:** Baseline characteristics of women filing a claim

	Total (*n* = 133)	Accepted claims (*n* = 54)	Rejected claims (*n* = 79)
Patient characteristics			
Age (years) (*n* = 133)	41 (15–77)	41 (25–68)	41 (15–77)
BMI (kg/m^2^) (*n* = 82)	25.0 (18.0–88.2)	24.9 (18.0–44.1)	25.7 (18.3–88.2)
ASA classification (*n* = 60)			
ASA 1	38 (63.3)	19 (70.4)	19 (31.7)
ASA 2	21 (35)	8 (29.6)	13 (21.7)
ASA 3 en 4	1 (1.7)	0	1 (1.6)
Previous surgery (*n* = 115)			
Laparotomy	46 (40.0)	23 (62.2)	23 (57.5)
Laparoscopy	31 (27.0)	14 (37.8)	17 (42.5)
Job (*n* = 97)			
Health care job	21 (21.6)	7 (15.9)	14 (26.4)
Parity (*n* = 118)			
0	30 (25.4)	13 (26.5)	16 (23.5)
1	20 (16.9)	6 (12.2)	14 (20.6)
>1	68 (57.7)	30 (61.3)	38 (55.9)
Number of claims from (*n* = 129)			
Teaching hospitals (27)	72 (55.8)	29 (55.8)	42 (55.3)
Non-teaching hospitals (36)	57 (44.2)	23 (44.2)	34 (44.7)
Type of surgery and main indication			
LH	26 (19.5)	11 (20.4)	15 (19.0)
Fibroids	17 (65.4)	7 (63.6)	10 (66.7)
Heavy menstrual bleeding	5 (19.2)	2 (18.2)	3 (20.0)
Malignancy	3 (11.6)	1 (9.1)	2 (13.3)
Endometriosis	1 (3.8)	1 (9.1)	0
Adnexal surgery (salpingectomy or cystectomy)	45 (33.8)	23 (42.6)	22 (27.8)
Cyst(s)	36 (53.5)	19	17 (77.4)
Adhesions	3 (4.3)	2	1 (4.5)
Suspected ovarian torsion	1 (4.3)	0	1 (4.5)
Suspected malignancy	1 (4.3)	0	1 (4.5)
Unknown	3 (13)	1	2 (9.1)
Diagnostic laparoscopy	25 (18.8)	9 (16.7)	16 (20.3)
Adhesions/chronic pain/	14 (57.7)	6 (75)	8 (50.0)
Infertility	7 (26.9)	3 (25)	4 (25.0)
Heavy menstrual bleeding	1	0	1 (6.3)
Acute abdominal pain	2 (7.6)	0	2 (12.3)
Staging ovarian tumor	1 (3.8)	0	1 (6.3)
Laparoscopic sterilization	21 (15.8)	6 (11.1)	15 (19.0)
Clips	7 (30.0)	2 (33.3)	5 (33.3)
Tuba cleavage	11 (52.4)	2 (33.3)	9 (60.0)
Unknown	1 (4.9)	1 (1.7)	0
Sterilization not performed	2 (5.7)	1 (1.7)	1 (6.7)
Other procedures	16 (12.0)	5 (9.3)	11 (13.9)
Adhesiolysis	5 (38.9)	4 (83.3)	1 (9.0)
Ectopic pregnancy surgery	4 (22.2)	1 (16.7)	3 (27.3)
IUD removal in abdomen	2 (11.1)	0	2 (18.2)
Laparoscopic sacrocolpopexy	5 (27.8)	0	5 (45.5)

Data are expressed as median (minimum–maximum) or as frequency (%)

*ASA* American Society of Anesthesia, *LH* laparoscopic hysterectomy, *IUD* intra-uterine device

**Fig. 1:**
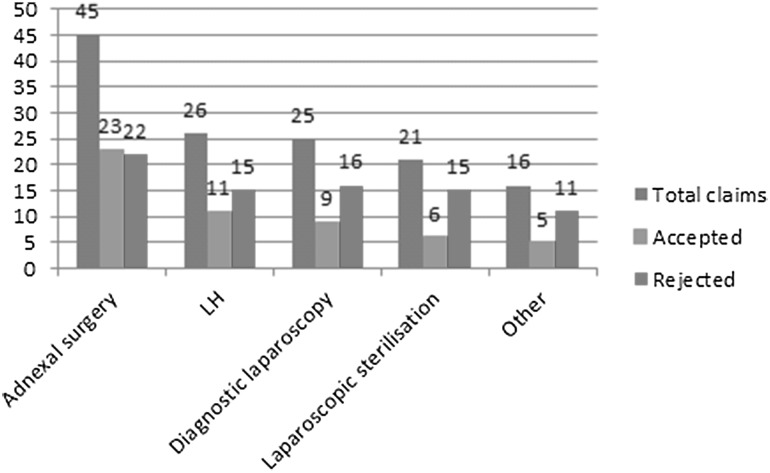
Claims per type of surgery

Figure 1 presents an overview of the claims stratified by type of surgery. Adnexal surgery was associated with the highest number of claims (33.8%), followed by laparoscopic hysterectomy (LH) (19.5%), diagnostic laparoscopy (18.8%), and laparoscopic sterilization (15.8%). The other procedures (12%) included adhesiolysis, ectopic pregnancy surgery, laparoscopic removal of an intra-uterine device in the abdomen, and laparoscopic sacrocolpopexy. Based on the classification of the ESGE^9^, 77% of the filed claims were non-advanced procedures (levels 1 and 2).

### Malpractices

**Fig. 2:**
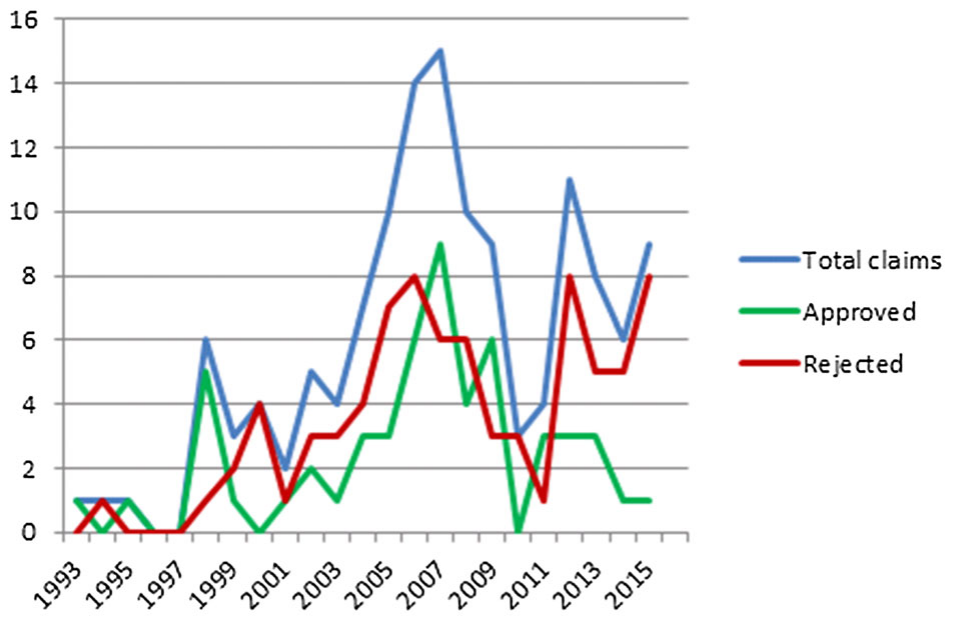
Overview of claims over the study period

Figure 2 demonstrates the total number of claims per year. On average, six claims were filed per year. The highest incidence of claims was observed in 2007 (15 claims). Our data showed that 91.7% of the claims related to LH were filed in the last 10 years (from 2005). No other specific trends were observed when stratifying the claims by type of procedure or type of injury (data not shown).

As can be observed in Table 2, 81.9% of the claims were filed for visceral and/or vascular injuries, and specifically 39.8% for bowel and 19.5% for ureter injuries. The bowel injuries were not related to a specific laparoscopic procedure, whereas 92% of the ureter injuries occurred during LH or adnexal surgery. In 51 claims (38.3%), including 19 accepted claims, the introduction of the needle and/or trocar caused the injury. It was not always explicitly mentioned in the medical files that the adverse events were entry related, but when evident we classified them into this group (e.g., diagnostic laparoscopy with artery iliac injury). The entry-related incidents caused in total 35 bowel injuries, nine vessel injuries, six bladder injuries, and one stomach injury (in a patient without nasogastric intubation). Twelve claims (9%), including five accepted ones, were filed for thermal injuries [bowel (*n* = 5), ureter (*n* = 6), and nerve (*n* = 1)]. These injuries were all discovered postoperatively and re-operation was required in all cases. Technical failure played a role in six cases (4.5%), of which all claims were approved. These technical failures were related to the inappropriate use of instruments (*n* = 1) and of laparoscopic monitor (*n* = 1). In the other four cases, surgical items were accidentally retained into the abdomen [needle (*n* = 2), sheath of instrument (*n* = 1), and gauze after conversion (*n* = 1)].

**Table 2:** Overview of the main type of claims and their severity

	Total (*n* = 133)	Accepted claims (*n* = 54)	Rejected claims (*n* = 79)
Type of injury			
Injuries	109 patients (81.9), 111 injuries	42 patients (75.9), 43 injuries	67 patients (78.5), 68 injuries
Bowel	53^a^ (39.8)	18^a^ (33.3)	35 (44.3)
Ureter	26^a^ (19.5)	13 (24)	13^a^ (16.5)
Bladder	13^a^ (9.7)	4^a^ (7.4)	9^a^ (11.4)
Vessel/hemorrhage	15 (11.3)	5 (9.3)	10 (12.7)
Stomach	1 (0.75)	1 (1.9)	0
Nerve	3 (2.2)	2 (3.7)	1 (1.3)
Chemical peritonitis	3 (2.2)	2 (3.7)	1 (1.3)
Wound dehiscence	4 (3.0)	1 (1.9)	3 (3.8)
Pulmonary embolism	1 (0.8)	0	1 (1.3)
Other	16 (12)	9 (16.6)	7 (8.9)
Unnecessary conversion	1	1	0
Skin burned	1	1	0
Foreign body	4	3	1
Failed procedure	4	0	4
Wrong procedure	4	4	0
Missed diagnose	1	0	1
Persistent symptoms	1	0	1
Cause of injury			
Laparoscopic entry-related	51 (38.3)	19 (35.2)	32 (40.5)
Thermal injury	12 (9.0)	5 (9.3)	7 (8.9)
Technical failure	7 (5.3)	6 (11.1)	1 (1.3)
No iatrogenic injuries	18 (13.5)	7 (13.0)	11 (13.9)
Unspecified	44 (33.1)	17 (31.5)	27 (34.2)
Severity of injury			
(A) Conservative treatment	19 (14.3)	10 (18.5)	9 (11.4)
(B) Re-intervention necessary	95 (71.4)	35 (64.8)	60 (75.9)
(C) Permanent disability	15 (11.3)	8 (14.8)	7 (8.9)
(D) Death	4 (3.0)	1 (1.9)	3 (3.8)
Moment discovered			
(1) Intra-operatively	26 (19.5)	14 (25.9)	12 (15.2)
(2) Postoperatively	40 (30.1)	14 (24.1)	26 (32.9)
(3) After discharge	67 (50.4)	26 (48.1)	41 (51.0)

Data are expressed as frequency (%)

^a^Two patients had two injuries

Concerning the severity of the injuries, 104 patients (78.2%) had to be re-operated at least once (including seven patients from category C and two from category D) and 84 of these patients had a laparotomy during re-operation (80.8%) (Table 2). In four patients, the adverse event resulted in death (3%). Three of these claims were rejected as no malpractice was observed. The first patient had a massive pulmonary embolism, the second one a massive hemorrhage during surgery for an initially suspected torsion of the ovary that appeared to be a sarcoma, and the third one died as a result of a sepsis after bowel injury diagnosed postoperatively. The fourth patient, whose case was accepted, died postoperatively as a result of sepsis after missed ureter injury. Her case was accepted because of delay in diagnosing the injury (exact time frame unclear). In 15 patients (11.3%), permanent disabilities occurred, including total loss of kidney function and nephrectomy after missed ureter injury, paralysis due to plexus lesions after malpositioning during surgery, or permanent stoma after bowel perforation. Half of all the injuries were discovered after discharge (50%). Specifically for the accepted claims, 89.5 and 91.7% of the bowel and ureter injuries, respectively, were missed intra-operatively. Almost all these patients had to be re-operated (94.7% of the bowel injuries and all ureter injuries).

### Legal information

The principle reason for approving a claim is depicted in Table 3: 18 claims (33.3%) were related to a delay in diagnosing the injury (postoperatively), 14 claims (25.9%) to negligence during surgery (operative skills, malpositioning during surgery, or wrong surgery), 11 claims (20.4%) to the consequences of the injury itself, and five claims (9.3%) to an incomplete informed consent. A wrong indication or an incomplete medical file played a role in 2 (3.7%) and 3 (5.6%) claims, respectively. In one claim (1.9%) the reason was unclear.

**Table 3:** Main reason for accepting a claim

	Accepted claims (*n* = 54)
Delayed/missed diagnosis or complication	18 (33.3)
Negligence during surgery	14 (25.9)
During operation	8
Malpositioning during surgery	2
Wrong surgery	4
Consequences of the event itself	11 (20.4)
Incomplete informed consent	5 (9.3)
Indication for surgery	2 (3.7)
Incomplete medical file	3 (5.6)
Unknown	1 (1.9)

Data are expressed as frequency (%)

Regarding the costs of the closed claims, the median total cost of the rejected claims was €374 (0–18094) and €14,569 (500–897,282) for the approved claims (Table 4). The total cost included all expenses made by the insurance companies, including the costs of, e.g., medical experts and attorneys as well as the direct financial compensation for the patients. The median sum directly paid to the patients and their attorneys was €12,000 (500–848,689). The highest pay-out was given to a woman who had a bowel injury after diagnostic laparoscopy because of chronic abdominal pain. Her claim was approved as the patient was not properly counseled about the risks and the choice for laparoscopic approach was disputed because of her medical history (history of perforated appendix complicated by an adhesion ileus).

**Table 4:** Financial and time overview of closed claims

	Total claims (*n* = 133)	Accepted claims (*n* = 54)	Rejected claims (*n* = 79)
Legal information (all claims, *n* = 133)			
Representative of interests	90 (67.6)	45 (83.3)	45 (57.0)
Civil procedure	14 (32.6)	6 (11.1)	8 (10.6)
Finances (in €) (closed claims, *n* = 125)			
Total sum	1560 (0–897,282)	14,569 (500–897,282)	374 (0–18,093.8)
Sum paid directly to patients	–	12,000 (500–848,689)	–
Time frame (days) (closed claims, *n* = 125)			
Incident to filing a claim	231 (5–2192)	218 (5–1999)	239 (12–2192)
Filing a claim to closure	661 (104–4064)	1219 (141–3960)	516 (104–4064)

Data are expressed as median (minimum–maximum) or as frequency (%)

An attorney was defending the patient in 90 claims (67.6%). For the accepted claims, 83.3% had an attorney compared to 57% for the rejected claims. Patients who were represented by an expert were 2.6 times (95% confidence interval 1.4–4.9) as likely as those without to receive financial compensation for their filed claims. The median time frame between the incident and the moment the patient filed a claim was 231 days (5–2192). From the moment the first complaint letter was sent out, it took a median period of 516 days (104–4064) to close the case for the rejected claims and 1219 days (141–3960) for the approved claims.

## Discussion

In an era where Value-Based Health Care is being broadly implemented, it is important not to focus only on surgical outcome measures to evaluate provided care but also to assess patient experience and outcome. In this line, data on medical claims provide a unique additional insight into incidents judged by patients as being substandard. Understanding the reasons for filing claims and sharing the data can be of added value for all practicing physicians.

Between 1993 and 2015, 133 claims were filed in The Netherlands after laparoscopic gynecologic procedures (six ρclaims per year on average). The claims were relatively equally distributed over time, except for two unexplained peaks in 2007 and 2012. Both insurance companies reported observing similar trends in other medical fields in those years without being able to further explain it. Although our data do not seem to show a specific trend over time, conclusions are difficult to draw as the total number of procedures performed over the study period is unknown. However, to put the numbers in perspective, a study by Twijnstra et al. demonstrated that in 2007, 16,863 laparoscopic gynecological procedures were performed in The Netherlands (response rate 80%) [10], while 15 claims were filed (0.09%). Furthermore, studies evaluating the implementation of laparoscopic gynecologic surgery demonstrated a significant increase in the number of laparoscopic procedures from 2002, 2007, and 2012 [10–12], and this was specifically the case for advanced surgeries (levels 3 and 4). From our medical claim data, no such trend was observed and therefore it seems that the wide expansion of laparoscopic surgery was not associated with an increase in medical claims. In the same line, it would be interesting to further study the relation between surgical experience and the number of claims. More than two decades of experience with advanced laparoscopic surgery does not seem to guarantee a decrease in the number of claims. But again, this should be stated with caution as the overall number of procedures performed in the study period has been increasing.

In 41% of the studied claims, financial compensation was granted. Compared to other (non-European) countries, The Netherlands has a high rejection rate and relatively low payments, but also a low threshold for filing a claim as not handled through a jury trial [13]. Similar to other European systems, financial compensation is in The Netherlands only granted if the event has been judged as being the consequence of medical negligence, i.e., that it could have been avoided. As a result, claims filed for severe consequences do not necessarily result in financial compensation. This was reflected in our study by the three cases of deceased patients whose families did not receive any financial compensation as the adverse events were judged as inherent risks related to the procedures.

Most claims in our study were provoked by injuries to the bowel and ureter. Bowel and ureter injuries are rare but are known to have a high morbidity, especially if diagnosed with substantial delay (e.g., thermal injuries) [14–17]. Overall, delay in diagnosing complications was the most reported reason for granting financial compensation (33%). This was in line with another claim study in general surgery that demonstrated that 26% of their 294 studied claims were related to delayed, wrong, or missed diagnosis [18]. In our study, patients with postoperative delayed diagnosis had often sought medical care (sometimes more than once) but because of the often unspecific symptom presentation of ureter and/or bowel injuries, injuries were not always (directly) recognized. Furthermore, it is important to realize that as the length of hospital stay after laparoscopic procedures is decreasing, most of these complications will only become manifest when patients are already at home. As a result, patients should receive sufficient instructions regarding the postoperative period and should be taken seriously when seeking care. Patients with (unspecific) symptoms, even a long time after surgery, need close monitoring until the diagnosis becomes clear or symptoms disappear [15, 17, 19].

A total of 51 claims (38%) were entry related. Wild et al. [20] demonstrated in their study that one-fifth of all laparoscopy-related claims in surgery were entry-related complications. Although specific risk factors, such as high BMI and previous procedures, have been associated with an increased risk of entry-related complications [20], needle and/or trocar insertion remains, in all patients and during all type of procedures, still one of the most hazardous steps in laparoscopy.

In the present study, 77% of the claims concerned non-advanced procedures (levels 1 and 2). It is important to realize that the denominators of the different procedures are unknown and therefore this finding does not imply that the incidences are necessarily higher for non-advanced laparoscopic procedures. Yet, it can be hypothesized that when an adverse event occurs in non-advanced procedures, it might be more difficult for a patient to accept it, as less expected. As a result, a detailed preoperative counseling is mandatory, even for routine procedures [21]. In The Netherlands, there are currently no government-mandated forms that must be used during counseling. It is the responsibility of the surgeons to adequately counsel their patients. It is self-evident that an incomplete informed consent weakens legal defense [5].This was observed in our study in five cases (9.3%), where financial compensation was primarily granted because of incomplete informed consent. Furthermore, we want to emphasize that it is important that residents are also aware of the possible impact of incomplete counseling. A slightly higher number of claims were filed by women treated in teaching hospitals and it cannot be excluded that the inexperience of residents in counseling but also regarding surgical skills did influence these results. Another interesting finding in our study was that 20% of the claims were filed by women working in the medical sector themselves. A potential explanation is that they have more medical knowledge and might, as a result, be more critical regarding the incident. Finally, 85% of the approved claims had an attorney, compared to 57% for the rejected claims (relative risk 2.6). Although bias by severity may have occurred, it seems that patients being represented by an expert have a higher chance of being financially compensated.

### Strengths and limitations

One of the limitations of this study was that 49 files (15%) of claims potentially meeting our inclusion criteria were destroyed. It is unclear though if all these claims would have been included in our study anyway: from our initial search, 146 (44%) did not meet our inclusion criteria either. Secondly, the data of the present study are based on the Dutch litigation system. Although the different European countries have overall similar liability laws, we are aware that our data might not be applicable to every country. Despite this limitation, we believe that our results provide an interesting overview of cases judged by patients as substandard care. Furthermore, this study was not conducted to provide an incidence number of adverse events, but rather to evaluate the type of filed claims. Finally, the largest proportion of claims originated from MediRisk. All claims from MediRisk insured hospitals are directly sent to the insurance company, whereas Centramed only gets involved when hospitals pay a starting fee. As a result, many claims from Centramed hospitals are handled in the initial hospital and these data were not available to us. Strengths of this work included the long study period and the fact that it provides a national overview (96% of the Dutch hospitals).

## Conclusion

Over the study period of more than 20 years, the number of claims remained relatively constant. Most claims were provoked by injuries to the bowel and ureters and most claims were filed after non-advanced laparoscopic procedures (77%). Entry-related complications accounted for 38% of the claims and delay in diagnosing injuries was the primary reason for granting financial compensation. Based on our findings, gynecologists are recommended to closely monitor their patients in the postoperative period and to give them specific instructions for the first weeks at home. Secondly, it is important to realize that entering the abdominal cavity during laparoscopy is still a potential dangerous first step. Therefore, for any type of laparoscopic procedure, doctors should take time to thoroughly counsel their patients, even regarding the risk of entry-related injuries.

## Electronic supplementary material

Below is the link to the electronic supplementary material.

Supplementary material 1 (DOCX 38 kb)

## References

[1] Sachdeva AK, Russell TR (2007). Safe introduction of new procedures and emerging technologies in surgery: education, credentialing, and privileging. Surg Clin N Am.

[2] https://www.rijksoverheid.nl/documenten/convenanten/2011/12/23/convenant-veilige-toepassing-van-medische-technologie-in-het-ziekenhuis. Accessed 5 Jun 2017

[3] https://www.rijksoverheid.nl/documenten/rapporten/2011/09/20/medische-technologie-at-risk. Accessed 5 Jun 2017

[4] de Vries EN, Eikens-Jansen MP, Hamersma AM, Smorenburg SM, Gouma DJ, Boermeester MA (2011). Prevention of surgical malpractice claims by use of a surgical safety checklist. Ann Surg.

[5] White AA, Pichert JW, Bledsoe SH, Irwin C, Entman SS (2005). Cause and effect analysis of closed claims in obstetrics and gynecology. Obstet Gynecol.

[6] Driessen SR, Baden NL, van Zwet EW, Twijnstra AR, Jansen FW (2015). Trends in the implementation of advanced minimally invasive gynecologic surgical procedures in The Netherlands. J Minim Invasive Gynecol.

[7] Darzi A, Mackay S (2002). Recent advances in minimal access surgery. BMJ.

[8] Istre O (2015). Minimally invasive gynecological surgery.

[9] Twijnstra AR, Zeeman GG, Jansen FW (2010). A novel approach to registration of adverse outcomes in obstetrics and gynaecology: a feasibility study. Qual Saf Health Care.

[10] Twijnstra AR, Kolkman W, Trimbos-Kemper GC, Jansen FW (2010). Implementation of advanced laparoscopic surgery in gynecology: national overview of trends. J Minim Invasive Gynecol.

[11] Kolkman W, Trimbos-Kemper TC, Jansen FW (2007). Operative laparoscopy in The Netherlands: diffusion and acceptance. Eur J Obstet Gynecol Reprod Biol.

[12] Driessen SR, Baden NL, van Zwet EW, Twijnstra AR, Jansen FW (2015). Trends in the implementation of advanced minimally invasive gynecologic surgical procedures in The Netherlands. J Minim Invasive Gynecol.

[13] de Reuver PR, Wind J, Cremers JE, Busch OR, van Gulik TM, Gouma DJ (2008). Litigation after laparoscopic cholecystectomy: an evaluation of the Dutch arbitration system for medical malpractice. J Am Coll Surg.

[14] Alkatout I, Schollmeyer T, Hawaldar NA, Sharma N, Mettler L (2012). Principles and safety measures of electrosurgery in laparoscopy. JSLS.

[15] Gilmour DT, Baskett TF (2005). Disability and litigation from urinary tract injuries at benign gynecologic surgery in Canada. Obstet Gynecol.

[16] Janssen PF, Brolmann HA, Huirne JA (2013). Causes and prevention of laparoscopic ureter injuries: an analysis of 31 cases during laparoscopic hysterectomy in The Netherlands. Surg Endosc.

[17] Llarena NC, Shah AB, Milad MP (2015). Bowel injury in gynecologic laparoscopy: a systematic review. Obstet Gynecol.

[18] de Vries EN, Eikens-Jansen MP, Hamersma AM, Smorenburg SM, Gouma DJ, Boermeester MA (2011). Prevention of surgical malpractice claims by use of a surgical safety checklist. Ann Surg.

[19] Janssen PF, Brolmann HA, Huirne JA (2011). Recommendations to prevent urinary tract injuries during laparoscopic hysterectomy: a systematic Delphi procedure among experts. J Minim Invasive Gynecol.

[20] Wind J, Cremers JE, van Berge Henegouwen MI, Gouma DJ, Jansen FW, Bemelman WA (2007). Medical liability insurance claims on entry-related complications in laparoscopy. Surg Endosc.

[21] Leclercq WKG, Keulers BJ, Scheltinga MRM, Spauwen PHM, van der Wilt GJ (2010). A review of surgical informed consent: past, present, and future. A quest to help patients make better decisions. World J Surg.

