# Supplementary material for: Medical malpractice claims in laparoscopic gynecologic surgery: a Dutch overview of 20 years
Source: Surg Endosc. 2017 Jun 20;31(12):5418–26. doi: 10.1007/s00464-017-5624-8 (PMC5715033; doi:10.1007/s00464-017-5624-8)
Supplement: Supplementary file 1 — Supplementary material 1 (DOCX 38 kb) [file 464_2017_5624_MOESM1_ESM.docx]

| **1^st^ level -- Basic level**   - Diagnostic laparoscopy - Sterilization - Needle aspiration of simple cysts - Ovarian biopsy |
| --- |
| **2^nd^ level -- Intermediate level**   - Salpingostomies for ectopic pregnancy - Salpingo-oophorectomies - Ovarian cystectomies - Adhesiolysis - Treatment of mild or moderate endometriosis-salpingostomy and salpingo-ovariolysis |
| **3dr level -- Advance level**   - Hysterectomy - Myomectomy - Treatment of incontinence - Surgery for severe endometriosis - Extensive adhesiolysis including bowel and ureter - Repair of simple intestinal or bladder injuries |
| **4^th^ level -- Procedures under evaluation or practiced in specialized centers**   - Pelvic floor defects - Oncology procedures: lymphadenectomy, radical hysterectomy and axiolloscopy - Rectovaginal nodules - Others |

**Appendix**

**Table S1:** ESGE classification of laparoscopic procedures

**Table S2:** Complication classification according to the NVOG

| Main category | Complication |
| --- | --- |
| Infection | - Local - Organ - Systemic |
| Injury | - Vascular - Bowel - Bladder - Ureter - Other |
| Wound dehiscence | -- |
| Hemorrhage | - > 1000 mL - Post-operative bleeding |
| Thrombo-embolism | -- |
| Dysfunction | - Urinary retention - Incontinence - Ileus - Liver - Kidney |
| Systemic | - Medication error - Adverse drug event - Other |
| Technical | - Failed procedure - Retained foreign body |
| Reactive conversion | -- |
| Other | -- |

**Figure S1:** Flow-chart of selected claims

**Exclusion n=146**Medi Risk **n=109,** Centramed **n=37**- Pregnancy after laparoscopic sterilisation (n=62)
- Intra-uterine procedures (n=26)
- Misclassification (n=37) (e.g. abdominal hysterectomy)
- Double (two claims for one same patient) (n=1)

Search from 1^st^ of January 1993 (MediRisk) and 1^st^ of January 1995 (Centramed) up to 31th of December 2015

TOTAL SEARCH **n=328**
MediRisk **n=257,** Centramed **n=71**

**Claims not found n=49**MediRisk **n=29,** Centramed **n=20**

Claims included **n=133**
MediRisk **n=119,** Centramed **n=14**Closed **n=117,** Open **n=16**
